# PD146 Can We Properly Evaluate Genetic And Genomic Applications? A Systematic Review Of Health Technology Assessment Reports

**DOI:** 10.1017/S0266462324003805

**Published:** 2025-01-07

**Authors:** Giuseppe Migliara, Antonio Sciurti, Ilaria Mussetto, Maria Roberta De Blasiis, Giuseppe Di Lorenzo, Dr Francesco Pierri, Immacolata Leone, Carolina Marzuillo, Paolo Villari, Valentina Baccolini

## Abstract

**Introduction:**

The last decade has witnessed a steady adoption of personalized medicine. However, the evaluation of genetic and genomic tests is not straightforward. The purpose of this systematic review was to identify health technology assessment (HTA) reports assessing genetic and genomic tests to summarize the methodologies used, the maturity level of the evidence included, and the highlighted research gaps.

**Methods:**

The PubMed, Scopus, and Web of Science databases were searched for HTA reports of genetic or genomic tests. The main national and international HTA report repositories (e.g., the international HTA database) were also searched. HTA reports that were specifically created to assess genetic or genomic technologies and included at least three core evaluation components (analytic validity, clinical validity, clinical utility, economic evaluation, organizational aspects, or ethical, legal, and social implications) were included. This study was supported by the European Commission and the Ministry for Universities and Research under the National Recovery and Resilience Plan (M4C2-I1.3 Project PE_00000019 “HEAL ITALIA”).

**Results:**

Overall, 27,331 unique records were retrieved, 55 of which were included in the systematic review. The reports were mainly from Australia (29%), Canada (27%), and the UK (25%); focused on pharmacogenomics (36%) and oncology (35%); and investigated test use for treatment guidance (42%) or diagnosis (29%). The most reported evaluation components were economic evaluation (87%), clinical utility (76%), and clinical validity (67%). On the other hand, personal utility (7%), patients’ perspectives (27%), and ethical (15%), legal (11%), and social (24%) implications were poorly represented. Analytical validity, safety, and organizational aspects were included in about half of the reports.

**Conclusions:**

Although these are only preliminary results, the substantial lack of a shared standard in the evaluation of genetic and genomic applications is clear given the heterogeneity of the dimensions addressed among the reports. Theres is a need to strengthen evaluation of the neglected dimensions, which are often of primary importance in defining the value and risks of personalized medicine.

